# The Role of TRPA1 Channels in the Central Processing of Odours Contributing to the Behavioural Responses of Mice

**DOI:** 10.3390/ph14121336

**Published:** 2021-12-20

**Authors:** János Konkoly, Viktória Kormos, Balázs Gaszner, Zoltán Sándor, Angéla Kecskés, Ammar Alomari, Alíz Szilágyi, Beatrix Szilágyi, Dóra Zelena, Erika Pintér

**Affiliations:** 1 Department of Pharmacology and Pharmacotherapy, Medical School, University of Pécs, H-7624 Pécs, Hungary; konkojani1@gmail.com (J.K.); viktoria.kormos@aok.pte.hu (V.K.); zoltan.sandor@aok.pte.hu (Z.S.); angela.kecskes@aok.pte.hu (A.K.); alomariammar75@gmail.com (A.A.); 2 Centre for Neuroscience, Szentágothai Research Centre of the University of Pécs, H-7624 Pécs, Hungary; balazs.b.gaszner@aok.pte.hu (B.G.); dora.zelena@aok.pte.hu (D.Z.); 3 Research Group for Mood Disorders, Department of Anatomy, Medical School, University of Pécs, H-7624 Pécs, Hungary; 4 Institute of Physiology, Medical School, University of Pécs, H-7624 Pécs, Hungary; aliz_szilagyi@yahoo.com (A.S.); szilagyibeatrix0621@gmail.com (B.S.); 5 Institute of Experimental Medicine, H-1085 Budapest, Hungary

**Keywords:** TRPA1, social behaviour, innate fear, piriform cortex, olfactory bulb, olfactory epithelium, 2-methyl-2-thiazoline, valeric acid

## Abstract

Transient receptor potential ankyrin 1 (TRPA1), a nonselective cation channel, contributes to several (patho)physiological processes. Smell loss is an early sign in several neurodegenerative disorders, such as multiple sclerosis, Parkinson’s and Alzheimer’s diseases; therefore, we focused on its role in olfaction and social behaviour with the aim to reveal its potential therapeutic use. The presence of *Trpa1* mRNA was studied along the olfactory tract of mice by combined RNAscope in situ hybridisation and immunohistochemistry. The aversive effects of fox and cat odour were examined in parallel with stress hormone levels. In vitro calcium imaging was applied to test if these substances can directly activate TRPA1 receptors. The role of TRPA1 in social behaviour was investigated by comparing *Trpa1* wild-type and knockout mice (KO). *Trpa1* mRNA was detected in the olfactory bulb and piriform cortex, while its expression was weak in the olfactory epithelium. Fox, but not cat odour directly activated TRPA1 channels in TRPA1-overexpressing Chinese Hamster Ovary cell lines. Accordingly, KO animals showed less aversion against fox, but not cat odour. The social interest of KO mice was reduced during social habituation–dishabituation and social interaction, but not during resident–intruder tests. TRPA1 may contribute to odour processing at several points of the olfactory tract and may play an important role in shaping the social behaviour of mice. Thus, TRPA1 may influence the development of certain social disorders, serving as a potential drug target in the future.

‡ These authors contributed equally as last authors.

## 1. Introduction

Transient receptor potential ankyrin 1 (TRPA1), a nonselective cation channel, may contribute to several physiological and pathological processes, including neurodegeneration. In our previous studies we provided substantial evidence that the lack of this ion-channel in *Trpa1* knockout (KO) animals decelerated the progression of diverse neurodegenerative processes (e.g., demyelination, fibre loss [1,2,3]), while the presence of TRPA1 ameliorated age-related memory decline [4]. Based on the role of TRPA1 receptors in the abovementioned pathological processes, we presume that this ion channel could be a promising therapeutic target in the treatment of these disorders. Smell loss is an early sign of neurodegeneration, therefore our study focused on the role of TRPA1 in olfaction.

**Figure 1:**
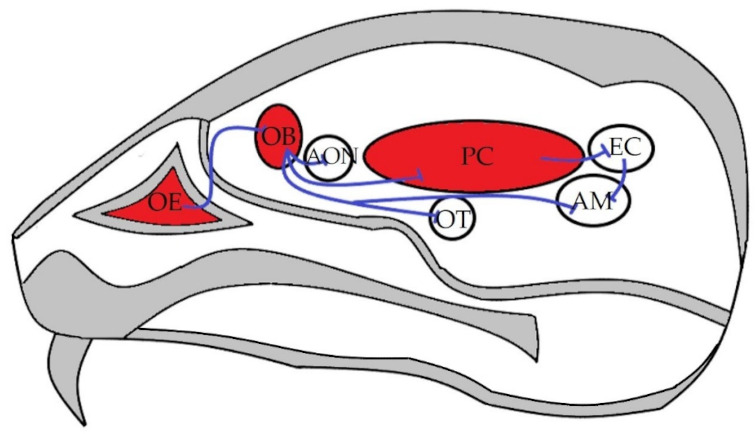
Schematic representation of the mouse olfactory tract. Red colour shows the investigated brain areas, Reprinted from ref [10]. Abbreviations: OE: olfactory epithelium, OB: olfactory bulb, AON: accessory olfactory nucleus, OT: olfactory tubercle, PC: piriform cortex, EC: entorhinal cortex, AM: amygdala.

The olfactory tract is one of the oldest among the principal sensory systems allowing smell. Olfaction begins in the olfactory epithelium (OE) located in the nasal cavity [5]. Its sensory bipolar neurons (OSN) have a peripheral dendrite which is responsible for odour sensation, and a central axon transmitting the stimuli—via glomerular synapses—to the glutamatergic cells of the olfactory bulb (OB) [5,6,7]. Another important cell type in the OB is the GABAergic granule cell, interacting with the neurotransmission of glutamatergic neurones [7]. The axon bundles of the glutamatergic cells form the olfactory tract and terminate in primary and secondary olfactory areas (i.e., anterior olfactory nucleus (AON), olfactory tubercle (OT), piriform cortex (PC), amygdala (AM) and entorhinal cortex (EC)) [8] (Figure 1). The largest primary olfactory area is the PC, which, after processing, transmits information to the secondary olfactory and association brain areas. Besides the above detailed main olfactory tract, the accessory olfactory system also conveys signals to higher-order centres contributing to behavioral responses, such as social interaction, in several mammalian species [9].

The anatomical structure of the human olfactory tract resembles that of rodents. However, olfaction is not essential for human survival and the olfactory system therefore plays a less important role in our daily life, being less developed compared to animals (e.g., rodents) [5]. Nevertheless, olfactory stimuli contribute to our social behaviour [11]; furthermore, in most mammals, they are important in establishing social hierarchies, recognition of mating partners, and caring for offspring [12,13,14,15]; although this information may remain unconscious, acting presumably through the limbic system. In rodents, sniffing behaviour may contribute to agonistic social behaviour towards the conspecific, conveying multiple types of information [16]. However, the sniffing behaviour of mice varies with behavioural context and can also be modulated by nonolfactory signals [17]. Olfactory signals influence the development of both short- and long-term social memory [18]. In turn, object recognition by rodents requires only intact vision [19,20].

Moreover, the functional integrity of the olfactory system is necessary for the detection of food sources and avoiding predators [12], and can therefore contribute to innate fear and anxiety as well. Predator-derived chemostimuli, leading to avoidance behaviours, can be driven through the activation of both the main and the accessory olfactory system. Using these chemostimuli, an increased neuronal activation has been detected within some limbic structures (e.g., AM), playing an important role in innate fear and anxiety [21]. In line with the above, an altered activation of PC, and the bed nucleus of the stria terminalis (BST)—together with increased norepinephrine release in the hippocampus—can be responsible for predator odour-evoked stress responses [22]. Interestingly, other authors could not detect enhanced innate fear using the same predator odour in naive mice [23].

A wide range of neurological diseases can be associated with olfactory disfunction, such as schizophrenia [24], bipolar disorder [25], major depression [26], and post-traumatic stress disorder [27]. Furthermore, anosmia is a typical symptom accompanying various neurodegenerative disorders [28,29]. The loss of smell may occur years before the onset of other symptoms of Parkinson and Alzheimer’s disorders, being the first hint for the development of neurodegenerative processes [30,31].

Several chemoreceptors participate in olfaction, among others, multiple transient receptor potential (TRP) cation channels [32]. One member of this superfamily is the TRP subfamily A member 1 (TRPA1), which contains a long ankyrin domain at its N-terminal, the cytoplasmic region being responsible for its thermal and chemical sensitivity [33]. TRPA1 is expressed in various tissues and can be activated by different endogenous and exogenous ligands. TRPA1 channels on the nociceptive afferents participate in the generation of pain signals under physiological and pathophysiological conditions (e.g., inflammation, chronic pain) [33]. This is supported by the fact that an autosomal dominant point mutation of this receptor leads to familial episodic pain syndrome with incapacitating upper body pain in infants [34]. In addition, the role of this receptor was also confirmed in neuropathic pain [35,36,37,38,39]. TRPA1 receptors are expressed on the trigeminal nociceptors contributing to the detection of irritating volatile agents with an aversive odour. Moreover, the genetic deletion of *Trpa1* led to a significant decrease in the emergence of innate fear in *Trpa1* knock out (KO) mice compared to their wild-type (WT) counterparts when using 2-methyl-2-thiazoline (2-MT), a compound imitating fox odour [40]. In this study, 2-MT induced lower neuronal activation in *Trpa1* KO animals—measured by c-Fos immunohistochemistry—in the trigeminal ganglia (TG) and the OB. Subsequently, enhanced neuronal activation was revealed in some stress-sensitive brain regions (periaqueductal grey matter (PAG) and paraventricular nucleus (PVN)), suggesting the role of TRPA1 in the activation of the hypothalamic–pituitary–adrenal axis, triggered by certain irritating compounds and mediated by TG and OB.

Here we aimed to reveal the expression of mouse *Trpa1* mRNA in the OE, OB and PC, the major parts of the olfactory tract. These brain areas are affected in the early stages of neurodegenerative disorders [29,31,41] explaining the emergence of anosmia even years prior to the onset of other neurological symptoms. To reveal the expression of *Trpa1* in the OE, OB and PC and determine the exact types of TRPA1-expressing cells, we performed RNAscope in situ hybridisation (ISH) combined with immunohistochemistry. To confirm the contribution of TRPA1 in predator odour-induced innate fear, we studied the receptor sensitivity towards the major components of fox (2-MT [40]) and cat odour (valeric acid [42,43]) in vitro using TRPA1-overexpressing CHO cells, and examined the behavioural influence of these two odours in WT and KO animals parallel with changes in stress hormone levels (adrenocorticotropin (ACTH) and corticosterone). Furthermore, to confirm the functional importance of TRPA1 channels in the olfactory tract, a detailed characterisation of the social behaviour of *Trpa1* KO and WT mice was also conducted.

## 2. Results

### 2.1. Trpa1 mRNA Expression and Colocalisation in the Piriform Cortex, Olfactory Bulb, and in the Olfactory Epithelium by RNAscope ISH

**Figure 2:**
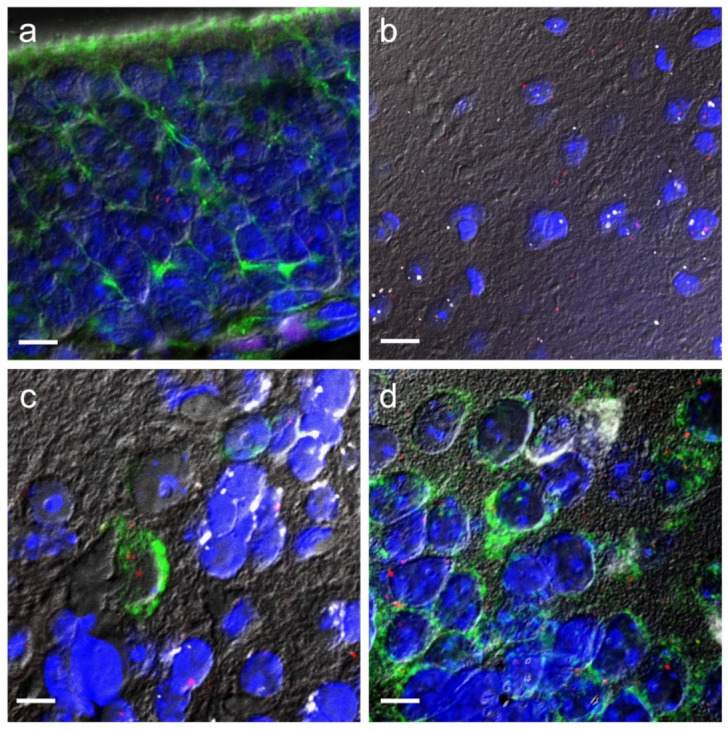
Expression of *Trpa1* mRNA in the investigated regions of the olfactory system of C57BL/6 mice (*n* = 4). *Trpa1* (red) mRNA signal did not colocalise with β-tubulin III (green) immunorective cells in the OE (**a**). *Trpa1* (red) mRNA signal colocalised exclusively with NeuN (white) positive neurons in the OB (**b**). *Trpa1* (red), Gad1 (white) and Vglut1 (green) mRNA expression in the OB (Bregma 3 mm), (**c**) and in the PC (**d**) (Bregma −1.46 mm). *Trpa1* mRNA signal colocalised both with Gad1 and Vglut1 positive neurons in the OB (**c**), but it colocalised only with Vglut1 positive neurons in the PC (**d**). Cell nuclei were counterstained with DAPI (blue) in all areas. Abbreviations: NeuN: neuronal nuclear protein, Gad1: glutamate decarboxylase 1, Vglut1: vesicular glutamate transporter 1, DAPI: 4′,6-diamidino-2-phenylindole. In order to highlight the cell borders, differential interference contrast (DIC) images were merged with the virtual color images. Bars: 10 µm.

To assess the possible presence of *Trpa1* in OSNs, RNAscope ISH for *Trpa1* mRNA was combined with anti-β-tubulin III immunohistochemistry, a selective marker of this cell type in the OE [44,45]. *Trpa1* mRNA was poorly detectable in the OE and did not colocalise with β-tubulin III (Figure 2a). In the OB, the *Trpa1* mRNA was colocalised in almost all cases with the neuronal marker, neuronal nuclear protein (NeuN), suggesting its almost exclusive neuronal presence (Figure 2b). In addition, using double RNAscope labelling, *Trpa1* mRNA was moderately expressed in glutamate decarboxylase 1 (Gad1) mRNA containing GABAergic neurons of the OB, but was poorly detected on glutamatergic excitatory neurons characterised by vesicular glutamate transporter 1 (Vglut1) (Figure 2c). In the PC, the RNAscope technique was sensitive enough to detect considerable amounts of *Trpa1* mRNA positive cells. In this region, the *Trpa1* mRNA was shown only on the Vglut1 mRNA containing excitatory neurons and did not colocalise with Gad1-positive cells (Figure 2d).

### 2.2. TRPA1 and Predator Olfaction

#### 2.2.1. Fox (2-MT) and Cat Odour (Valeric Acid) Induced Calcium Influx in Mouse and Human TRPA1-Overexpressing CHO Cells

**Figure 3:**
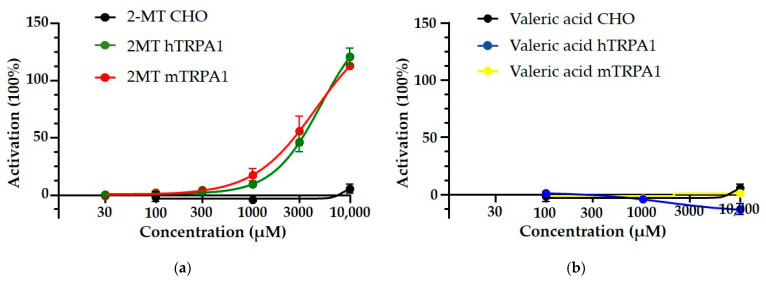
Effect of fox (2-MT) and cat odour (valeric acid) on the calcium response in Chinese Hamster Ovary (CHO) cells expressing human and mouse TRPA1 (hTRPA1 and mTRPA1, respectively) receptors. An increased calcium response was characterised by an enhanced ratio of Fluo-4 AM fluorescence compared to dye-loaded unstimulated cells. 2-MT resulted in a concentration-dependent elevation of the calcium response with an EC_50_ value of 5010 μmol/L in human TRPA1-expressing cells and with an EC_50_ value of 4419 μmol/L in mouse TRPA1-expressing cells (**a**). Applying valeric acid, neither human nor mouse TRPA1-expressing cell lines showed an elevated Ca^2+^ signal (**b**). No change of the calcium response was detected in CHO cells not expressing TRPA1 in response to 100, 1000 and 10,000 μmol/L 2-MT or valeric acid. *n* = 5–6 × 10^4^ cells for all types of cell lines, all experiments were performed four times.

Human and mouse TRPA1 channel-overexpressing Chinese Hamster Ovary cells (CHO) (*n* = 5–6 × 10^4^ cells per data point) showed a concentration-dependent increase in the green fluorescence ratio compared to dye-loaded unstimulated cells in response to 2-MT (fox odour), both in the case of mouse and human receptor expressing cells, without significant differences between the receptor activation of the two species (Figure 3a). The EC_50_ value was 5010 μmol/L in human and 4419 μmol/L in mouse TRPA1-expressing cells. CHO cells not expressing TRPA1, used as a negative control, did not respond to 2-MT. Similarly, neither TRPA1-expressing cell lines responded to valeric acid (cat odour) (Figure 3b).

#### 2.2.2. Interest of *Trpa1* WT and KO Animals towards Fox (2-MT) and Cat Odour (Valeric Acid)

**Figure 4:**
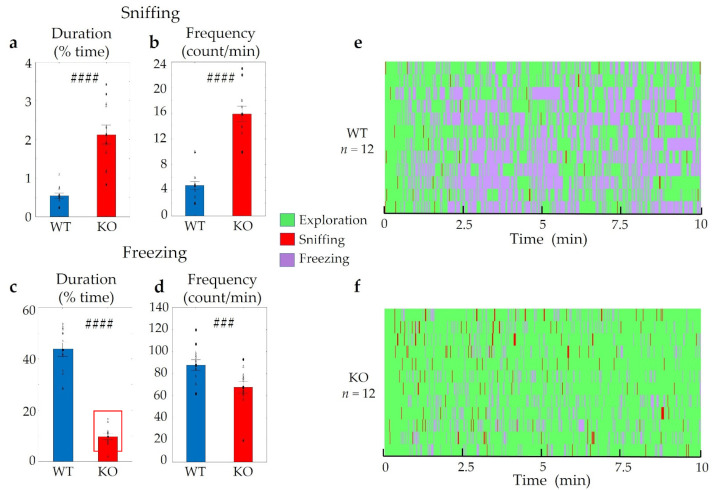
Behavioural differences in *Trpa1* wild-type (WT) and knockout (KO) mice triggered by the fox odour, 2-MT. Remarkable differences were established in the duration and frequency of sniffing the odour holder (**a**,**b**) and freezing behaviour (**c**,**d**) between the two groups. The Gantt diagram presents the individual animals (**e**,**f**). *n* = 12 in both (WT and KO) groups, the symbol # shows a significant difference between the two genotypes, in cases with ### *p* < 0.001, and #### *p* < 0.0001. Blank triangles represent individual values while dark spots show outliers (characterized by a higher or lower value than mean ± 2 standard deviation (SD)).

Considerable differences were detected between WT and KO mice during the 2-MT evoked odour aversion test. Both the duration and the frequency of sniffing the odour holder was significantly higher in *Trpa1* KO mice compared to WT animals (t_duration_ (2,23) = −6.52, *p* < 0.01; t_frequency_ (2,23) = −8.43, *p* < 0.01) (Figure 4a,b). On the other hand, freezing lasted for a significantly shorter period and occurred much less frequently in KO mice (t_duration_ (2,23) = 11.78, *p* < 0.01; t_frequency_ (2,23) = 2,81, *p* < 0.01) (Figure 4c,d).

**Figure 5:**
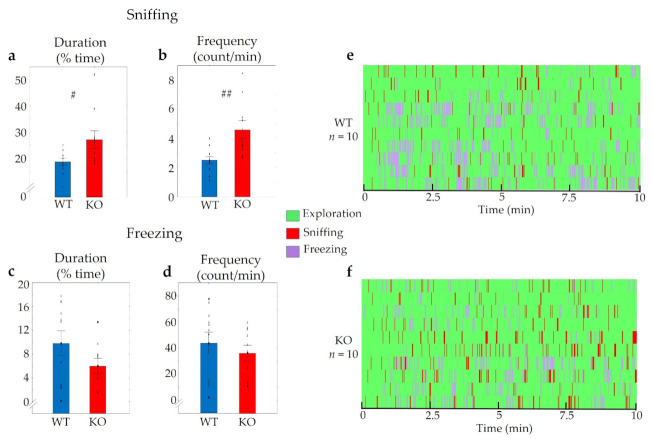
Behavioural differences in *Trpa1* WT and KO mice triggered by the major component of cat odour, valeric acid. Significant differences were detected in the duration (**a**) and frequency (**b**) of sniffing the odour holder between the two groups. However, differences in innate fear (freezing) were not present in this trial (**c**,**d**). Individual values are represented on a Gantt diagram (**e**,**f**). *n* = 10 in both (WT and KO) groups, the symbol # shows a significant difference between the two genotypes, in cases with # *p* < 0.05, and ## *p* < 0.01. Blank triangles represent individual values while dark spots show outliers (characterized by a higher or lower value than mean ± 2 standard deviation (SD)).

In the case of valeric acid, similar differences were observed between the genotypes in sniffing (t_duration_ (2,19) = −3.27, *p* < 0.01; t_frequency_ (2,19) = −2.40, *p* < 0.03) (Figure 5a,b), while only a tendentious difference was observed for freezing (p_frequency_ > 0.50, p_duration_ > 0.14) (Figure 5c,d). During this experiment, we investigated the temporal alterations of the frequency and duration of sniffing and found that KO animals sniffed the filter paper more often (F_genotype_ (1,18) = 4.44, *p* < 0.05; F_interaction_ (9,162) = 2.11, *p* < 0.04) and for a longer time (F_genotype_ (1,18) = 9.29, *p* < 0.01; F_interaction_ (9,162) = 1.96, *p* < 0.05) predominantly at the beginning and end of the examination (p_2fr_ < 0.04, p_5fr_ < 0.01, p_7fr_ < 0.03, p_8fr_ < 0.05 and p_2dur_ < 0.03, p_5dur_ < 0.01, p_7dur_ < 0.01, p_8dur_ < 0.01, p_10dur_ < 0.04) compared to their WT mates (Figure 5e,f). However, neither in the case of frequency nor duration, was any significant effect of time detectable (F_time frequency_ (9,162) = 0.49, *p* > 0.88; F_time duration_ (9,162) = 1.02, *p* > 0.42).

#### 2.2.3. Hormone (ACTH and Corticosterone) Measurements in WT and KO Mice after Using 2-MT or Valeric Acid

**Figure 6:**
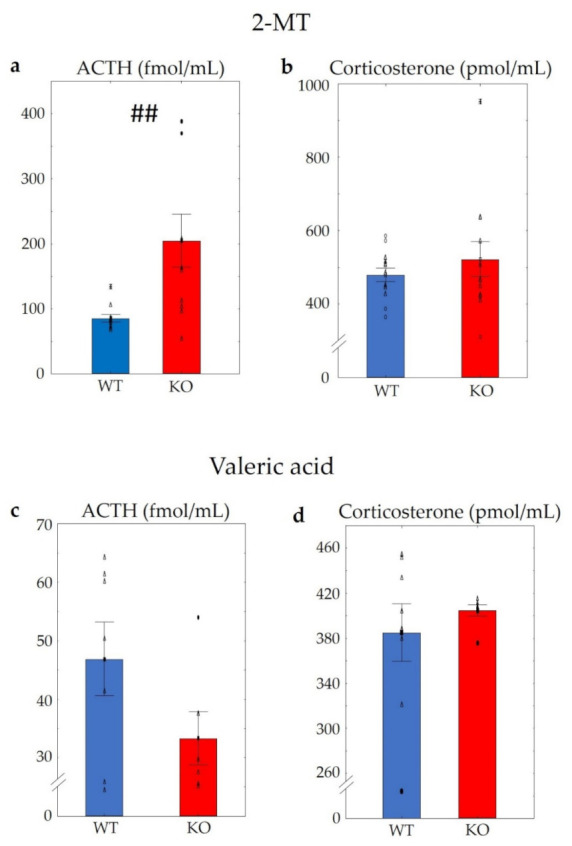
Serum adrenocorticotropin (ACTH) and corticosterone levels of WT and KO mice after using 2-MT or valeric acid. Applying 2-MT, KO mice showed significantly higher ACTH levels than WTs (**a**), without significant differences in the corticosterone levels (**b**). Using valeric acid, no differences in either ACTH (**c**) or in corticosterone levels (**d**) were detectable. The symbol # shows a significant difference between the two genotypes, in cases with ## *p* < 0.01. *n* = 10 in both (WT and KO) groups. Blank triangles represent individual values while dark spots show outliers (characterized by a higher or lower value than mean ± 2 standard deviation (SD)).

The investigation of blood samples at the end of a 10 min odour exposure revealed significantly higher ACTH levels in KO mice after using 2-MT (t (1,19) = −3.02, *p* < 0.01) (Figure 6a). However, differences in corticosterone levels were not established [t (1,23) = −0.85, *p* > 0.4] (Figure 6b). Morever, after the odour aversion test with valeric acid, no differences in either ACTH (t (1,11) = 1.69, *p* > 0.11) or in corticosterone levels (t (1,13) = −0.71, *p* > 0.49) could be detected (Figure 6c,d).

### 2.3. TRPA1 and Social Behaviour

#### 2.3.1. Object Habituation–Dishabituation and Social Habituation–Dishabituation Tests in WT and KO Mice

**Figure 7:**
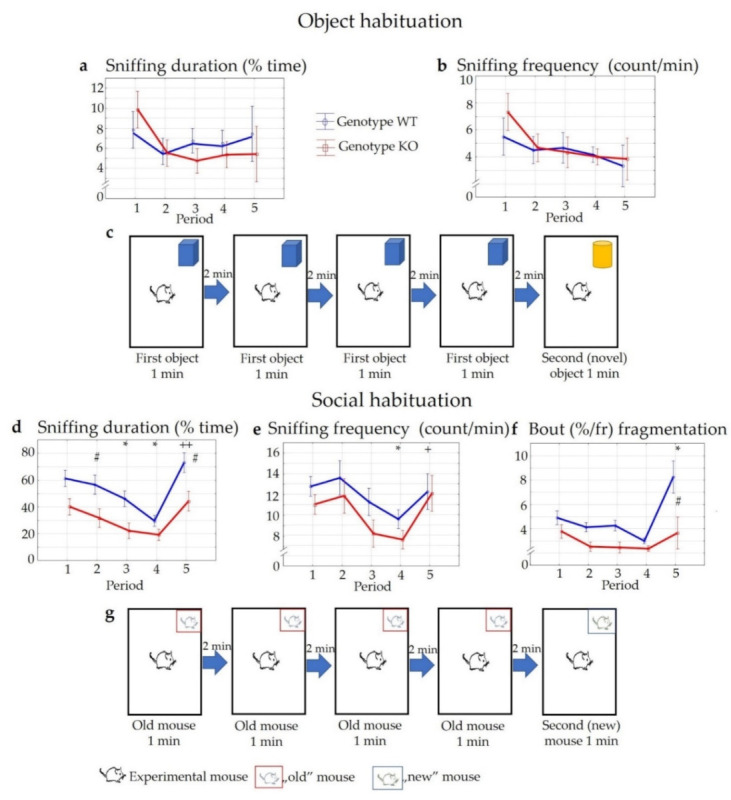
Results of object (**a**–**c**) and social habituation–dishabituation trials (**d**–**g**). There was no difference in the duration and frequency of sniffing an object between the genotypes during the object habituation test (**a**,**b**). During the social habituation trial, a remarkable decrease in the social behaviour was shown using the first stimulus repeatedly in mice of both genotypes. However, after adding a novel stimulus for mice, the social interactions were again increased (**d**–**f**). The social behaviour of KO mice was lower than WT mice during the whole examination period, with significant differences in the 2nd and 5th part of the trial (**d**). *n* = 12 in both groups, the symbol # shows a significant difference between KO and WT mice (*p* < 0.05); * shows a significant difference between the periods compared to the 1st part of the trial in the same group, in cases with * *p* < 0.05; + shows a significant difference between the 4th and the 5th part of the trial in the same group, in cases with + *p* < 0.05 and ++ *p* < 0.01.

No significant differences were detected in the duration or frequency of sniffing during the object habituation–dishabituation trial within or between the groups (duration: F_time_ (4,40) = 1.17, *p* > 0.33; F_genotype_ (1,10) = 0.32, *p* > 0.58; F_interaction_ (4,40) = 0.46, *p* > 0.76 and frequency: F_time_ (4,40) = 2.00, *p* > 0.11; F_genotype_ (1,10) = 0.17, *p* > 0.68; F_interaction_ (4,40) = 0.32, *p* > 0.86), either when the same object was presented repeatedly, or when a new object was introduced (Figure 7a,b).

In contrast, the social stimulus was more attractive than the object. Accordingly, the duration of social interactions was higher in both groups during the first encounter with the (“old”) stimulus mice but reduced rapidly during subsequent presentations (F_time_ (4,88) = 15.48, *p* < 0.01) (Figure 7d). This decrease in the social behaviour was significant during the third and fourth encounter in the case of KO animals (*p* < 0.05) and during the fourth encounter in the case of WT animals (p_4_ < 0.01). On the other hand, a remarkable increase in social interest was detectable using novel stimulus mice during the fifth period of the examination (*p* < 0.01 in both genotypes). The abovementioned alterations were present in both genotypes; however, the level of the social behaviours was lower in the KO mice during the whole experiment (F_genotype_ (1,22) = 12.61, *p* < 0.01; F_interaction_ (4,88) = 1.04, *p* > 0.39). A post hoc comparison revealed a significant genotype difference during the second and the fifth period of the trial (*p* < 0.05). Similar temporal alterations were detected in the case of the frequencies of the social interactions (F_time_ (4,88) = 4.49, *p* < 0.01), and these differences were significant during the fourth and fifth encounter in both genotypes (*p* < 0.05), although significant differences could not be established between WT and KO animals (F_genotype_ (1,22) = 2.02, *p* > 0.16; F_interaction_ (4,88) = 0.39, *p* > 0.82) (Figure 7e). On the other hand, the length of one sniffing period (bout fragmentation; duration/frequency) was also significantly lower in KO animals (F_genotype_ (1,22) = 14.10, *p* < 0.01; F_time_ (4,88) = 7.30, *p* < 0.01) and this effect was especially visible when new stimulus mice were introduced (F_interaction_ (4,88) = 2.71, *p* < 0.05) (Figure 7f). The level of aggression was very low (occurred an average of one time during the 1 min interaction) and without genotype differences.

#### 2.3.2. “Three Chamber” Sociability Experiment in WT and KO Animals

**Figure 8:**
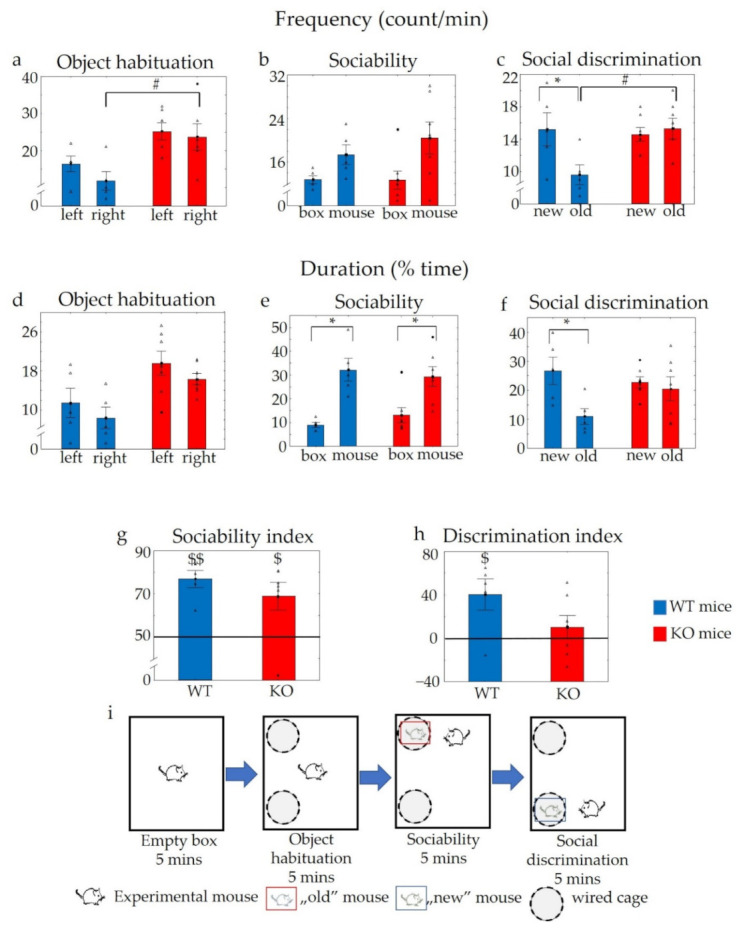
Results of the sociability test. During the object habituation phase, the KO animals investigated the objects more times than the WTs (**a**), but the difference in the duration was not significant (**d**). During the sociability phase, increased interest was detected towards the social stimulus in both groups without significant genotype differences (**b**,**e**). During the social discrimination phase, WT animals demonstrated increased interest towards the novel stimulus mice, but this kind of difference were not detectable in KO mice (**c**,**f**). Both genotypes revealed significant social interest as represented by a sociability index higher than 50% (**g**). However, only WTs showed intact short-term social memory as represented by a discrimination index higher than 0 (**h**). Schematic representation of the sociability trial (**i**). *n* = 5 in WT and *n* = 7 in KO groups, the symbol # shows a significant difference between KO and WT mice (*p* < 0.05); * shows a significant difference between the two sides with wired cages, in cases with * *p* < 0.05; $ shows a significant difference in the same group using a single sample t-test, in cases with $ *p* < 0.05, and $$ *p* < 0.01. Blank triangles represent individual values while dark spots show outliers (characterized by a higher or lower value than mean ± 2 standard deviation (SD)).

During the habituation phase of a subsequent sociability test, KO animals investigated the objects more frequently than their counterparts (F_genotype_ (1,10) = 8.9, *p* < 0.02), without a significant side preference (F_side_ (1,10) = 3.65, *p* > 0.08) and there was no genotype effect detectable on the side preference (F_interaction_ (1,10) = 0.41, *p* > 0.53) (Figure 8a). In the case of the investigation duration, the main significant effects of genotype and side preference were detected (F_genotype_ (1,10) = 7.41, *p* < 0.03; F_side_ (1,10) = 6.32, *p* < 0.04), although a post hoc comparison did not reveal significant differences (*p* > 0.05 in all cases), and the genotype did not influence the side preference (F_interaction_ (1,10) = 0.01, *p* < 0.92) (Figure 8d).

During the sociability (third) phase, all animals showed a higher duration of interest towards the social stimulus (first stimulus mice) (F_effect of choice_ (1,10) = 21.23, *p* < 0.01], without significant differences between the two genotypes (F_genotype_ (1,10) = 0.07, *p* > 0.79; F_interaction_ (1,10) = 0.65, *p* < 0.43). Moreover, there was no significant difference in the frequency of interest between the two genotypes (F_genotype_ (1,10) = 0.41, *p* > 0.53; F_interaction_ (1,10) = 0.59, *p* > 0.45), although the main effect of choice was significant (F_effect of choice_ (1,10) = 9.24, *p* < 0.02; in a post hoc comparison p_WT_ > 0.13 and p_KO_ > 0.08) (Figure 8b,e). A single sample t-test showed a remarkable social interest in the sociability index of both groups (t_WT_ (1,4) = 6.70, *p* < 0.01; t_KO_ (1,6) = 2.98, *p* < 0.05) when compared to the 50% chance level (Figure 8g).

During the social discrimination phase (last part) WT mice sniffed the novel stimulus animals significantly more often than the old counterparts (F_interaction_ (1,10) = 7.06, *p* < 0.02) (Figure 8c). However, there was no difference in the frequency of interest towards the new stimulus animals between the two genotypes (F_genotype_ (1,10) = 2.77, *p* > 0.13; F_effect of choice_ (1,10) = 4.23, *p* > 0.06). On the other hand, KO mice showed a significantly higher interest towards the old stimulus animals compared to the WT genotype (*p* < 0.04 in the post hoc comparison). In addition, WT mice dealt with the novel stimulus mice for a significantly longer time (F_effect of choice_ (1,10) = 6.21, *p* < 0.04; in the post hoc comparison *p* < 0.05), while a similar tendency in the duration of sniffing could not be detected in KO animals (*p* > 0.66) (Figure 8f). With respect to this parameter, there were no differences between the two groups (F_genotype_ (1,10) = 0.65, *p* > 0.44; F_interaction_ (1,10) = 3.49, *p* > 0.09). No direct differences in the sociability index (preferring the mice containing box above the empty one during the third phase of the test) and discrimination index (preferring the new stimulus animal above the old one during the fourth phase of the test) were detectable between the genotypes (p_SI_ > 0.36 and p_DI_ > 0.12). However, using a single sample *t*-test, only the WT mice showed an intact memory (t_WT_ (1,4) = 2.78, *p* < 0.05) (discrimination index > 0) and such an effect was not shown in KO animals (*p* > 0.38) (Figure 8g,h).

#### 2.3.3. Social Interaction and Resident–Intruder Trials in WT and KO Mice

**Figure 9:**
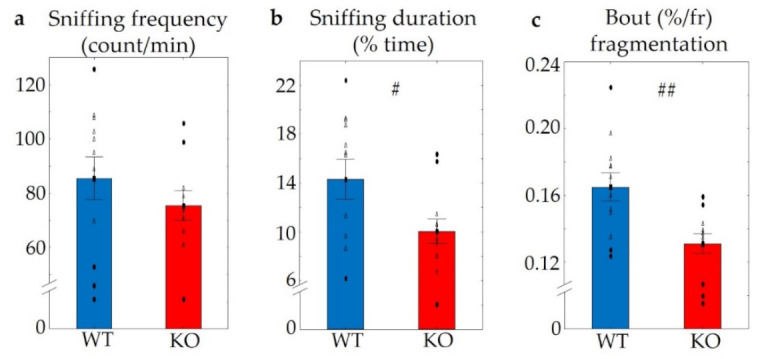
Results of the social interaction test. No differences were detected in the frequency of social behaviour between the two genotypes (**a**); however, a decreased duration of sniffing (**b**) and bout fragmentation (**c**) were detected in KO mice. *n* = 12 in both groups, the symbol # shows a significant difference, in cases with # *p* < 0.05, and ## *p* < 0.01. Blank triangles represent individual values while dark spots show outliers (characterized by a higher or lower value than mean ± 2 standard deviation (SD)).

During the social interaction test, differences in the sniffing frequency, representing friendly social interaction, were not detectable between the genotypes (t (2,22) = 1.06, *p* > 0.3) (Figure 9a), but we observed a significantly reduced time spent sniffing a conspecific (t (2,22) = 2.25, *p* < 0.04) (Figure 9b) and a decreased sniffing bout length (t (2,22) = 3.26, *p* < 0.01) (Figure 9c) in KO mice. On the other hand, we could not detect aggressive interactions during this experiment.

**Figure 10:**
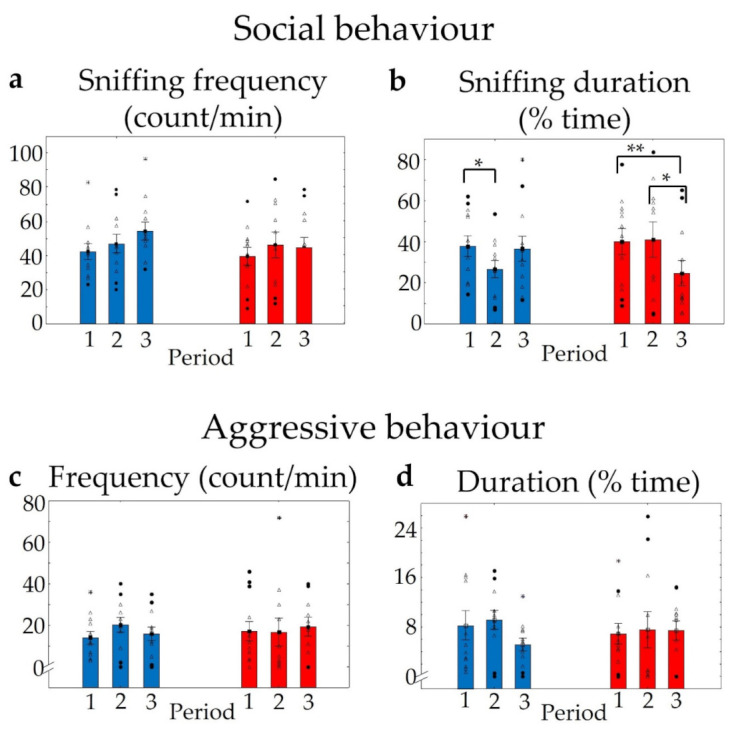
Results of resident–intruder trials. Numbers 1,2,3 represent 10 min trials 1 week apart. No significant differences were found in the frequency of social behaviour between the two genotypes (**a**). The duration of sniffing decreased significantly in both groups during the subsequent trials, although relevant temporal differences were detectable only in KOs (**b**). Neither the frequency nor the duration of aggressive interactions were significantly altered by time or by genotype (**c**,**d**). *n* = 12 in both groups, * *p* < 0.05 and ** *p* < 0.01 show a significant difference between repeated trials in the same group. Blank triangles represent individual values while dark spots show outliers (characterized by a higher or lower value than mean ± 2 standard deviation (SD)).

During repeated resident–intruder trials one week apart, we evaluated home cage defence, and no alterations were detected in the frequency of social behaviour within and between the two genotypes (F_genotype_ (1,21) = 0.50, *p* > 0.48; F_time_ (2,42) = 1.07, *p* > 0.35; F_interaction_ (2,42) = 0.36, *p* > 0.7) (Figure 10a), but the duration of friendly social behaviour was decreased significantly in both groups by repeating the trials (F_time_ (2,42) = 2.62, *p* > 0.08; in a post hoc comparison p_WT and KO 1–2 period_ < 0.05, p_KO 2–3 period_ < 0.01; and F_interaction_ (2,42) = 5.14, *p* < 0.01), although genotype differences could not be detected (F_genotype_ (1,21) = 0.11, *p* > 0.74] (Figure 10b). In addition, there was no significant temporal alteration in aggression (F_time duration_ (2,42) = 1.18, *p* > 0.31; F_time frequency_ (2,42) = 0.86, *p* > 0.43), as well as no detectable genotype effect (F_genotype duration_ (1,21) = 0.002, *p* > 0.96 and F_genotype frequency_ (1,21) = 0.07, *p* > 0.79) and the genotype did not influence the temporal changes (F_interaction duration_ (2,42) = 1.67, *p* > 0.2; and F_interaction frequency_ (2,42) = 1.91, *p* > 0.16) (Figure 10c,d).

## 3. Discussion

Here, we demonstrated the presence of *Trpa1* mRNA in various parts of the olfactory system with a prominent expression in the OB, colocalised with GABAergic and glutamatergic markers, and in the PC, with glutamatergic cells. In accordance with previous results, we confirmed that the TRPA1 channel is an important player of innate fear induced by a predator odour [40]. Our in vitro results, using TRPA1-overexpressing CHO cell lines revealed that only fox, but not cat odour is able to directly activate TRPA1 receptors in the OE and may contribute to a subsequent triggering of avoidance behaviour. Moreover, we also found that TRPA1 is a significant player in friendly, but not aggressive social interest and intact social memory, as in its absence, reduced social investigation was found during social habituation–dishabituation and social interaction tests and there was a loss of memory during the social discrimination phase of the sociability test.

It has already been suggested that fox odour is a direct activator of chemosensory TRPA1 channels in the OE [40]. Indeed, during the evaluation of Ca^2+^ influx in TRPA1-expressing CHO cells, we found that both the mouse and the human receptors respond to 2-MT. However, in our case the effective concentration was higher than previously described [40]. These differences might be explained by the different cell lines, and by the different experimental conditions. On the other hand, valeric acid could not induce any responses on either receptor. Indeed, valeric acid is a chemical compound belonging to the group of carboxylic acids and does not have an electrophilic structure, suggesting that it cannot directly influence TRPA1 receptors. Interestingly, KO mice showed slightly different behaviour to cat odour compared to their WT pairs. Although in the case of cat odour (valeric acid) a genotype difference was detected, but only in the interest towards the odour holder, not in freezing, and this might be explained by the overall less fearful nature of the cat odour. (In the case of fox odour this behavioural pattern accounts for around 45% of the entire time in WT mice and 10% in KO mice, while in case of cat odour around 10% in WT and 5% in KO animals.) Thus, we cannot exclude the possibility that the freezing reduction in KOs is masked by this low “basal” level. Despite its contribution to the behavioural consequences of predator odour, the lack of TRPA1 does not reduce fear responses in general. For example, in the case of conditioned fear, an amygdala–hippocampal–prefrontal cortex-dependent paradigm [46], *Trpa1* KO mice showed enhanced rather than reduced freezing [47]. Thus, we might assume that the interest towards an odour stimulus can be mediated by TRPA1 receptors through the olfactory system, more specifically in its central part, e.g., in the OB and PC, as the OE does not contain a considerable amount. As the chemical particles of the odour cannot directly reach the secondary/tertiary neurones, the TRPA1-induced alteration in sniffing time and frequency will be independent from the odour type. However, it must be also considered, that TRPA1 agonists (like fox odour) may lead to the activation of trigeminal nociceptors in the nasal epithelium, triggering local pain responses [48], which may also strongly influence the behavioural responses of the animals. Pain itself as well as a direct connection between the PC and PVN [49] may enhance the neuronal activity of stress-sensitive brain areas (e.g., PAG, PVN) [40] and certain limbic structures (e.g., hippocampus, BST) [22]. Controversially, the *Trpa1* KO animals reacted to 2-MT with higher ACTH levels, without any genotype differences in the corticosterone levels. This observation may be the consequence of their increased motility resulting from their less intense innate fear responses. Previous studies confirmed a connection between locomotion and ACTH levels [50]. In contrast to our findings, in another study the application of 2-MT induced a lower corticosterone elevation of KO animals compared to their WT counterparts [40]. This discrepancy can be explained by the different concentration of 2-MT used as well as by the distinct duration of the trials which might have covered the previously observed difference. More importantly, after applying valeric acid, none of the abovementioned stress hormones showed differences between the genotypes. This fact further confirms that the sensation of different odours may activate different pathways and that TRPA1 channels might influence this process at different places (e.g., in the case of fox odour, the trigeminal ganglion (pain) can activate the stress axis, while in the case of cat odour the OB/PC pathway might be involved in shaping the behaviour of the animals).

In support of this behavioural role of central TRPA1 receptors, a decreased social interest of KO mice was detected in the social habituation–dishabituation and social interaction trials. In agreement with this, they had a worse social memory (see discrimination index). Although we might assume that the social behaviour of KO animals might be impaired due to their higher distress triggered by a novel environment, their more frequent object approach contradicts this idea. Indeed, KO mice were more interested in a newly introduced object (see object habituation phase of sociability as well as the first encounter of the object in habituation–dishabituation, although this later effect was not significant, probably due to the short, 1 min observation time), which excludes the general loss of interest of *Trpa1* KO mice as well. This further supports the less anxious phenotype of KO mice [47]. However, their social behaviour was more fragmented suggesting an enhanced arousal [51]. In contrast to our results Lee et al. in 2017 found an enhanced social interest in *Trpa1* KO mice during an experiment similar to our sociability test. Although in their case the enhanced interest towards the container might have confused the results as there was no object habituation phase. Moreover, we used juvenile stimulus mice to avoid any possible threat from them, which might also influence the quality of the social interaction. Notwithstanding the above, we did not find any remarkable differences between the genotypes in aggressive behaviour, both during the social interaction as well as the resident–intruder trials. This implies that TRPA1 participates in the fine tuning of social behaviour and might regulate only certain aspects, especially friendly social interactions. We might assume that its role in olfaction might be an important player in its contribution to social behaviour. In support, although previous results found a better memory function of KO mice during novel object recognition tasks [47], during the social memory test the performance of this genotype was worse than its WT counterparts. Furthermore, it must be underlined that the recognition of objects in rodents is rather reliant on visual cues [19,20].

In support of the importance of TRPA1 in olfaction, we confirmed its presence at different levels of the olfactory system.

Nevertheless, *Trpa1* mRNA was expressed with only a low number of copies in the OE, and it was not colocalised with anti-β-tubulin III, a selective marker of OSNs [44,45], suggesting that the receptor may be expressed in other OE structures (e.g., on the ending of trigeminal neurons or in type B cells) [52]. The synthesis of this TRPA1 receptor protein may happen predominantly in the perikaryon of TG neurons, where *Trpa1* expression has been confirmed previously [53,54,55], contributing to the development of diverse (patho)physiological conditions [48]. The OB might be a major site of action of aversive chemostimuli as we detected a considerable amount of *Trpa1* mRNA here by RNAscope ISH. We detected *Trpa1* predominantly in GABAergic neurones, but some transcripts in glutamatergic neurons were also seen. In a previous study, *Trpa1* mRNA was also detected in the OB using RT-PCR, and further investigations confirmed its expression predominantly in glutamatergic mitral and GABAergic granule cells [56]. We confirmed for the first time that *Trpa1* is expressed also in the PC. Here, in the PC, *Trpa1* mRNA is expressed only in *Vglut1* positive glutamatergic neurons. These cells are known to be principal neurones receiving signals from other olfactory structures (e.g., OB), certain limbic brain areas (e.g., basolateral AM) and from several other parts of the brain. The diverse afferentation of these cells contributes to shaping information encoding [57]. These results suggest the relevance of TRPA1 receptors in the central processing of olfactory information.

In the central nervous system, TRPA1 was found on astrocytes [58,59] and oligodendrocytes [60]. The functional inhibition of TRPA1 located on oligodendrocytes may decrease the myelin damage caused by ischemia [60]. Moreover, the lack of TRPA1 attenuates the process of demyelination and oligodendrocyte apoptosis in cuprizone-treated mice, suggesting its role in the progression of autoimmune multiple sclerosis as well [1,2]. On the other hand, the presence of TRPA1 is necessary for normal neuronal development and oligodendrocyte maturation, and its functional ablation may lead to impaired emotion, cognition, learning, memory, and social behaviour [47]. TRPA1 is also increasingly considered as a major participant in the development and progression of certain neurodegenerative disorders [3]. TRPA1 may facilitate the synaptic dysfunction triggered by oligomeric amyloid-β peptide, implying its role in Alzheimer’s disease [61]. Intracerebral amyloid beta_1-42_ injection induced smaller cholinergic fibre loss in the somatosensory cortex accompanied by normalised memory function in *Trpa1* KO mice [3]. In addition, TRPA1 deficiency resulted in an attenuated memory loss in aged mice, suggesting the importance of this receptor in age-related memory decline as well [4]. In addition, our experiments confirmed the expression of TRPA1 receptors in the OB and the PC, which are brain areas that are already involved in the early stages of neurodegenerative disorders [29,31,41]. A possible mechanism of neurodegeneration may be the activation of TRPA1 channels leading to an increased calcium influx, triggering neuronal activation on one hand and in the case of excessive function, neuronal apoptosis. This process may occur in the glutamatergic excitatory neurons of the OB and PC and—together with consecutive glutamatergic excitotoxicity—may explain the appearance of anosmia in neurodegenerative diseases (e.g., Parkinson’s, Alzheimer’s) [62,63,64] 5–10 years prior to the emergence of other neurological symptoms [30,31].

In summary, our results confirmed the presence of TRPA1 at multiple levels of the olfactory tract playing an important role in the perception of aversive/irritating as well as social odours. This receptor, located in the central structures of the olfactory tract, may have a pivotal role in the central processing of odour information contributing to the association of emotional responses and behavioural patterns. Therefore, this ion channel could be a drug target in the treatment of diseases with disturbed emotions and social behaviour. In addition, TRPA1 receptors, located in the OB and PC, could be a promising target in the early treatment of certain neurodegenerative conditions by decelerating neuronal apoptosis in these brain areas.

## 4. Materials and Methods

### 4.1. Animals

RNAscope in situ hybridisation was performed using naive 9-week-old male C57BL/6 mice. Behavioural experiments were carried out on 3–4 months-old male *Trpa1*^+/+^ (wild-type, WT) and *Trpa1*^−/−^ (knockout, KO) mice. The original breeding pairs of mice were acquired from Prof. P. Geppetti, University of Florence, Italy. Mice were generated and characterised as described earlier [65]. Animals were bred on a C57BL/6J background and crossed back after 5 generations. The genotype of offspring for the *Trpa1* gene was verified by PCR. Four-week-old *Trpa1* WT (sociability, social habituation–dishabituation) or CD1 mice (resident–intruder test) were used as stimulus animals. All efforts were made to minimise the number of animals used and their suffering.

Animals were kept in a temperature and humidity controlled 12 h light–dark cycle environment (lights on at 6 a.m.) in standard polycarbonate cages (365 mm × 207 mm × 144 mm) at the animal facility of the Department of Pharmacology and Pharmacotherapy, University of Pécs. Ad libitum standard rodent chow and tap water were provided for the animals. Four to six mice were housed in a cage, and 1 week before the first resident–intruder test they were separated to have a “homecage”. The social behaviour of the animals was examined during the early dark phase between 19 and 23 h, while all other examinations were conducted in the morning.

### 4.2. RNAscope ISH and Immunohistochemistry

#### 4.2.1. Tissue Sample Collection and Preparation

The intact C57BL/6 male mice were deeply anaesthetised by intraperitoneal urethane injection (2.4 g/kg) and transcardially perfused with 20 mL of ice-cold 0.1 M phosphate-buffered saline (PBS, pH: 7.4) followed by 150 mL 4% paraformaldehyde (PFA) solution in Millonig buffer (pH 7.4) for 15 min. After perfusion, the brains with OBs were removed, and collected into PFA for 36 h for postfixation at 4 °C. Olfactory epithelia were also removed and collected into 4% PFA containing 30% sucrose for 36 h postfixation, to ensure cryoprotection. The brains with the OBs were coronally sectioned using a Leica VT1000 S vibratome (Leica Biosystems, Wetzlar, Germany). Five series of 30 µm sections were gathered and stored in antifreeze solution (20% ethylene glycol, 30% glycerol and 0.1 M sodium-phosphate buffer) at −20 °C. OE samples were embedded in OCT tissue freezing medium (Leica), and five series of 17 µm transversal sections were obtained using a cryostat (Leica CM1950, Nussloch, Germany). Then, sections were taken on SuperFrost Ultra Plus adhesion slides (Thermo Fisher Scientific, Braunschweig, Germany, Cat. No.: 10417002), and stored at −20 °C.

#### 4.2.2. RNAscope ISH on Mouse Olfactory Bulb and Piriform Cortex

The RNAscope assay was carried out on coronal OB and PC sections, applying RNAscope Multiplex Fluorescent Reagent Kit v.2 (Advanced Cell Diagnostics, Newark, CA, USA) according to the protocol of the product with a modified pretreatment [66]. Briefly, after the tissue pretreatment, samples were hybridised with probes specific to mouse *Trpa1* (Cat. No.: 400211-C2), *NeuN* (neuronal nuclear protein; Cat. No.: 313311-C3), *Vglut1* (vesicular glutamate transporter 1; Cat. No.: 416631) and *Gad1* (glutamate decarboxylase 1; Cat. No.: 400951-C3, Advanced Cell Diagnostics, Newark, CA, USA) mRNA. We performed sequential signal amplification and channel development according to the manual. 4**′**,6-diamidino-2-phenylindole (DAPI (Cat. No.: 323108, Advanced Cell Diagnostics, Newark, CA, USA)) was used to detect cell nuclei. Sections were cover-slipped with ProLong Diamond Antifade Mountant (Thermo Fisher Scientific) for confocal microscopy. To provide reliable results, we simultaneously applied a RNAscope 3-plex mouse positive control probe (ACD; Cat. No.: 320881, Advanced Cell Diagnostics, Newark, CA, USA) specific to RNA polymerase II subunit A mRNA (*Polr2a* (fluorescein)), peptidylprolyl isomerase B mRNA (*Ppib* (cyanine 3, Cy3)) and ubiquitin C mRNA (*Ubc* (cyanine 5, Cy5)) and 3-plex negative control probes (ACD; Cat. No.: 320871) to bacterial D-box binding PAR BZIP transcription factor (*dabP)* mRNA (Supplementary Materials Figure S1). To obtain fluorescent images of the OB and PC, an Olympus Fluoview FV-1000 laser scanning confocal microscope and FluoView FV-1000S-IX81 image acquisition software system (Olympus, Tokyo, Japan) were used. The confocal aperture was set to 80 µm. We conducted the analogue sequential scanning with a 40 × objective lens (NA: 0.75). We applied an optical thickness of 1 µm and the resolution was set to 1024 × 1024 pixels. The excitation time was set to 4 µs per pixel. The following virtual colours were used for the fluorescent signals: blue for DAPI, green for fluorescein (488 nm) (*Vglut1* mRNA), red for Cyanine 3 (550 nm) (*Trpa1* mRNA), and white for Cyanine 5 (647 nm) (*Gad1* and *NeuN* mRNA). Images were contrasted using ImageJ software (version 1.52a, NIH). To establish the colocalisation of the *Trpa1* mRNA signal with the *NeuN, Gad1* and *Vglut1* mRNA signal as well as with the immunohistochemistry signal of anti-β-Tubulin III, we performed a differential interference contrast (DIC) technique combined with the fluorescence signal.

#### 4.2.3. RNAscope ISH Combined with β-Tubulin III-Immunohistochemistry

We also performed RNAscope ISH on transversal sectioned OE according to the above detailed protocol. Subsequently, β-tubulin III immunohistochemistry was also conducted to mark OSNs. Briefly, after channel development of the RNAscope assay, sections were washed for 2 × 15 min in PBS, incubated overnight at RT with polyclonal rabbit anti-β-tubulin III (Merck, Sigma Aldrich GmbH; Cat. No.: T2200, Schnelldorf, Germany), diluted 1:200 with 2% normal donkey serum for blocking aspecific binding. Then, sections were washed for 2 × 15 min in PBS, and treated in Alexa Fluor 488-conjugated donkey anti-rabbit secondary antibody (A-11012, Thermo Fisher Scientific), diluted 1:500 in 1 × PBS with 2% normal donkey serum for 3 h at RT. After rinses, cell nuclei were counterstained with DAPI, and the sections were mounted with ProLong Diamond Antifade Mountant for confocal microscopy.

### 4.3. Measurement of Ca^2+^ Influx in TRPA1-Expressing CHO Cells in Response to 2-MT or Valeric Acid by Flow Cytometry

Cell lines overexpressing mouse and human TRPA1 were constructed according to the protocol used in the Department of Pharmacology and Pharmacotherapy, University of Pécs, Medical School [67]. Culture medium (500 mL Dulbecco’s-Modified Eagle Medium (DMEM), 50 mL fetal bovine serum albumin, 10 mL L-glutamine (200 mmol/L), 10 mL MEM non-essential amino acid solution, 500 µL penicillin and streptomycin) was gently removed from cells and a trypsin solution (250 µL, 0.1% in PBS) was applied for 5 min. For each sample approximately 10^4^ TRPA1-expressing CHO cells were resuspended in 100 μL cell culture medium. Fluo-4 AM (Invitrogen, 0.4 µL, 1 µg/µL in DMSO) was added for 30 min at 37 °C. Extracellular solution (ECS) was added (400 µL, containing (in mmol/L): NaCl, 160; KCl, 2.5; CaCl_2_, 1; MgCl_2_, 2; HEPES, 10; glucose, 10; pH 7.3). An appropriate amount of 2-MT or valeric acid was added to the cell suspensions in 500 μL ECS. Cell suspensions were analysed by flow cytometry. Fluo-4 AM was excited by a 488 nm laser. Fluorescence was detected at 504 nm. The mean green fluorescence of the samples was compared to the base fluorescence of dye-loaded control cells and to receptor carrying cells activated with 100 μM of the TRPA1 agonist, allyl-isothiocyanate, representing 100% activation [67].

### 4.4. Behavioural Tests

The behaviour of the animals was video recorded and later scored by computer-based event-recorder software (Solomon coder https://solomon.andraspeter.com/, 8 August 2019) by an experimenter blinded to the treatment groups. The following behavioural patterns were investigated: inactivity/freezing (no obvious activity), exploration (walking through the box or sniffing towards the environment), social investigation (sniffing at partner) and aggression (wrestling with the intruder often accompanied by biting or pushing down the opponent while it is trying to escape). Both the duration (in percentage) and frequency of all behaviours were registered. Exploration and sniffing were analysed in all trials, aggression was investigated in the social interaction and resident–intruder test, but freezing was only registered in the odour aversion tests.

#### 4.4.1. Odour Aversion Tests and Blood Sample Collection

**Figure 11:**
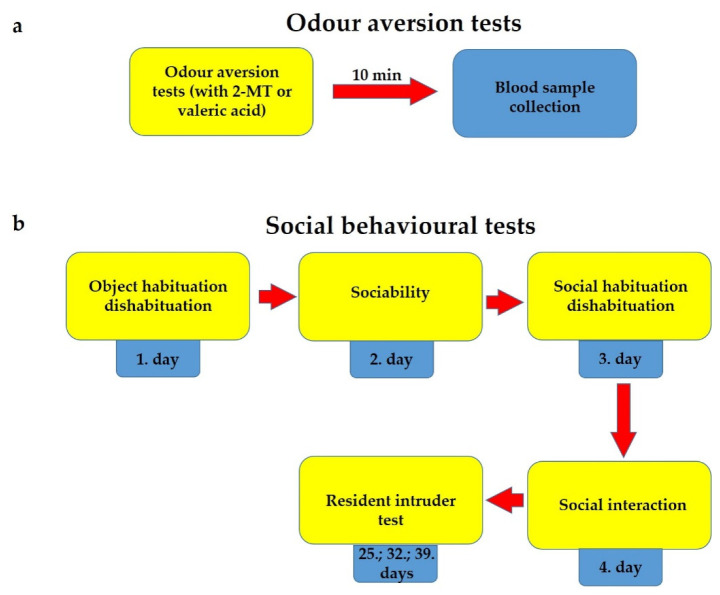
Schematic representation of the process of odour aversion (**a**) and social behavioural experiments (**b**), carried out on *Trpa1* knockout mice and their wild-type siblings.

The experiment was performed in a transparent box located in a sound-attenuated room within a hood with a camera above the box. In the corner of the box a tube was fixed, containing a filter paper scented with 2-MT (Sigma-Aldrich, Cat. No.: 2346-00-1) or valeric acid (Merck, Sigma Aldrich GmbH, Schnelldorf, Germany, Cat. No.: 800821). The substances were diluted with saline (50 μL to 2 mL) and a 50 μL sample was used for each animal. The mice were allowed to freely explore the arena for 10 min. The boxes were cleaned with ethanol between animals and the odour was refreshed using a new filter paper and odour. Right at the end of this trial, animals were sacrificed, blood was collected into prechilled tubes, and the samples were centrifuged at 3000 rpm for 5 min. Supernatant serum samples were collected and stored at −20 °C for ACTH and corticosterone radioimmunoassay experiments (Figure 11).

#### 4.4.2. Test Battery for Social Behaviour

##### Day 1: Object Habituation–Dishabituation

The animals were put in the test cage with nontransparent walls, filled with fresh bedding. After a 2 min habituation, an object was added to the box for four 1-min long trials with an inter-trial interval of 2 min. During the fifth trial a novel object was used with a different colour and shape. Objects were inserted in the corner of the box in all cases, but the location of the corner was randomised. The objects were small plastic toys (built from coloured plastic cubes and having complex shapes). Prior to the experiment all items were cleaned with ethanol (Figure 7c).

##### Day 2: Sociability Test

The experiment consisted of four trials, all of them lasted for 5 min. For the first five minutes the experimental animals were put in the empty box, to acclimatise to the new environment. During the second phase, two empty wired cages were placed into the corner of the box for 5 min, to habituate the animals to these objects (habituation). During the third period a stimulus animal was placed below one of the wired cages (social interest), and here, we calculated the sociability index (in this index the interest towards the social partner is divided by the sum of the interest towards the box and social partner). During the last five minutes a novel stimulus mouse was inserted below the second wired cage (social discrimination), and we calculated the discrimination index with the following formula: (new mouse − old mouse)/(old + new mouse) × 100 (Figure 8i).

##### Day 3: Social Habituation–Dishabituation

The experimental design was similar to that of the object habituation protocol with the sole difference, that in this trial, 4-week-old *Trpa1* WT stimulus animals were used. Briefly, a *Trpa1* WT stimulus male mouse was placed into the box for four 1-min long unrestricted interactions with an inter-trial interval of 2 min. During the intervals, the stimulus mouse was housed alone in a holding container and the experimental animal remained in the testing cage. In the fifth phase of the trial, a novel stimulus animal was used from a different cage (Figure 7g).

##### Day 4: Social Interaction

This test was also performed during the dark period for the animals; however, the investigating room was lighted to enhance the anxiety component. Two experimental animals, belonging to the same group but living in different cages, were placed into the same box for 10 min, and the interactions between them were registered.

##### Resident–Intruder Test

Resident–intruder trials were performed on days 25, 32 and 39 after at least 4 days of single housing to ensure that the test animal considered the cage as its home. On the day of the trial a smaller 4-week-old CD1 mouse (intruder) was inserted into the cage for 10 min.

### 4.5. Hormone Measurement

Hormone levels were measured by radioimmunoassay in unextracted serum (ACTH: 50 μL; corticosterone: 10 μL), both using a specific antibody developed at the Institute of Experimental Medicine (Budapest, Hungary) [68]. The intra-assay coefficients of variation were 7.5% and 4.7%, respectively. All samples from a particular experiment were measured in one session.

### 4.6. Statistical Analysis

Data are represented as mean ± SEM with individual data as dark spots. The comparison between WT and KO groups was conducted by paired sample *t*-test. Main effects were studied by factorial or repeated-measures ANOVA as shown in the text. A post hoc analysis was carried out by the Fisher and Tukey test depending on the number of investigated groups. For social and discrimination indices, the single sample *t*-test was used in comparison to 50% (social) or 0% (discrimination). All statistical analyses were performed using Statistica 13.5.0 software. Datasets were tested for normal distribution and for homogeneity of variance. If the *p*-value was lower than 0.05, it was considered statistically significant.

## 5. Conclusions

We confirmed the presence and role of the TRPA1 receptor in olfaction and—in this context—in the regulation of aversive and social behaviour. Thus, TRPA1 might be a promising drug target for the treatment of certain behavioural disorders. Furthermore, the location of TRPA1 in the primary olfactory cortex—and the possibility of neuronal apoptosis induced both by calcium and by glutamatergic excitotoxicity [69,70] due to the excessive activation of the receptor—may explain the occurrence of anosmia, as an early marker, in several neurodegenerative disorders. Thus, TRPA1 targeting drugs may decelerate neuronal apoptosis, and prevent the further development of the disorders at an early stage.

## Data Availability

Data is available within the article and supplementary material.

## Supplementary Materials

The following are available online at https://www.mdpi.com/article/10.3390/ph14121336/s1. Figure S1: Electrophoretograms of RT-PCR products. Six olfactory bulb samples of C57BL/6 mice were investigated. The housekeeping gene (glyceraldehyde 3-phosphate dehydrogenase (*Gapdh*), size: 237 bp) and the gene of interest (*Trpa1*, size: 101 bp) were expressed in all samples (a). Eight piriform cortex samples of C57BL/6 mice were investigated. The housekeeping gene (*Gapdh*, size: 237 bp) was expressed in all samples but a detectable level of gene of interest (*Trpa1*, size: 101 bp) could be found only in three cases (b). NTC: no template control; noRT: no reverse transciptase controls. Figure S2: RNAscope triplex positive and negative controls in the mouse piriform cortex. RNAscope triplex negative control probes would hybridize with the bacterial *dabP* gene (a). RNAscope triplex positive control probes specific to mouse *Polr2a* (b), *Ppib* (c) and *Ubc* (d). mRNA targets are represented in green, red and white, respectively. Abbrevations: *dabP*: D-box binding PAR BZIP transcription tactor, *Polr2a*: RNA polymerase II subunit A, *Ppib*: peptidylprolyl isomerase B, *Ubc*: ubiquitin C.
