# Supplementary material for: The Role of TRPA1 Channels in the Central Processing of Odours Contributing to the Behavioural Responses of Mice
Source: Pharmaceuticals (Basel). 2021 Dec 20;14(12):1336. doi: 10.3390/ph14121336 (PMC8703823; doi:10.3390/ph14121336)
Supplement: Supplementary file 1 [file pharmaceuticals-14-01336-s001.zip › pharmaceuticals-1504580-supplementary.pdf]

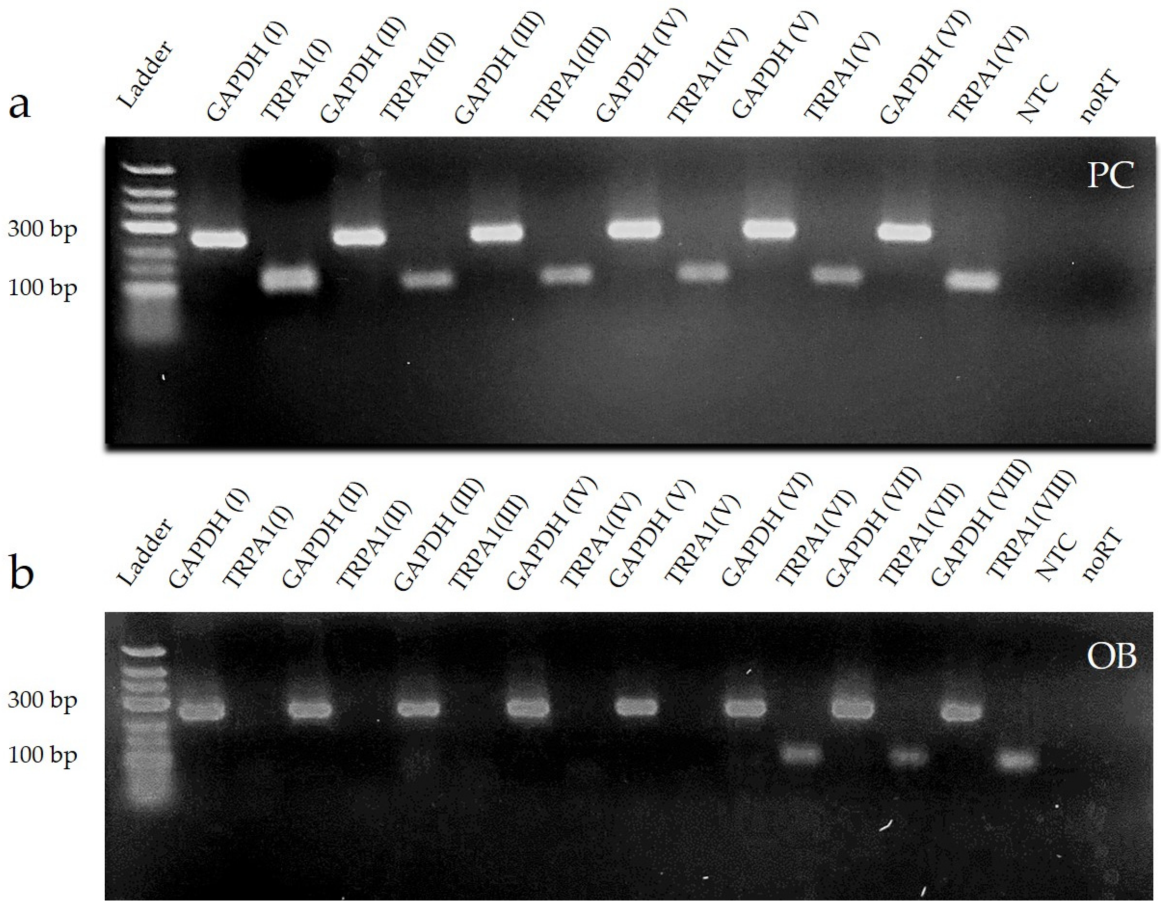

Figure S1. Electrophoretograms of RT-PCR products. Six olfactory bulb samples of C57BL/6 mice were investigated. The housekeeping gene (glyceraldehyde 3-phosphate dehydrogenase (*Gapdh*), size: 237 bp) and the gene of interest (*Trpa1*, size: 101 bp) were expressed in all samples (**a**). Eight piriform cortex samples of C57BL/6 mice were investigated. The housekeeping gene (*Gapdh*, size: 237 bp) was expressed in all samples but a detectable level of gene of interest (*Trpa1*, size: 101 bp) could be found only in three cases (**b**). NTC: no template control; noRT: no reverse transciptase controls.

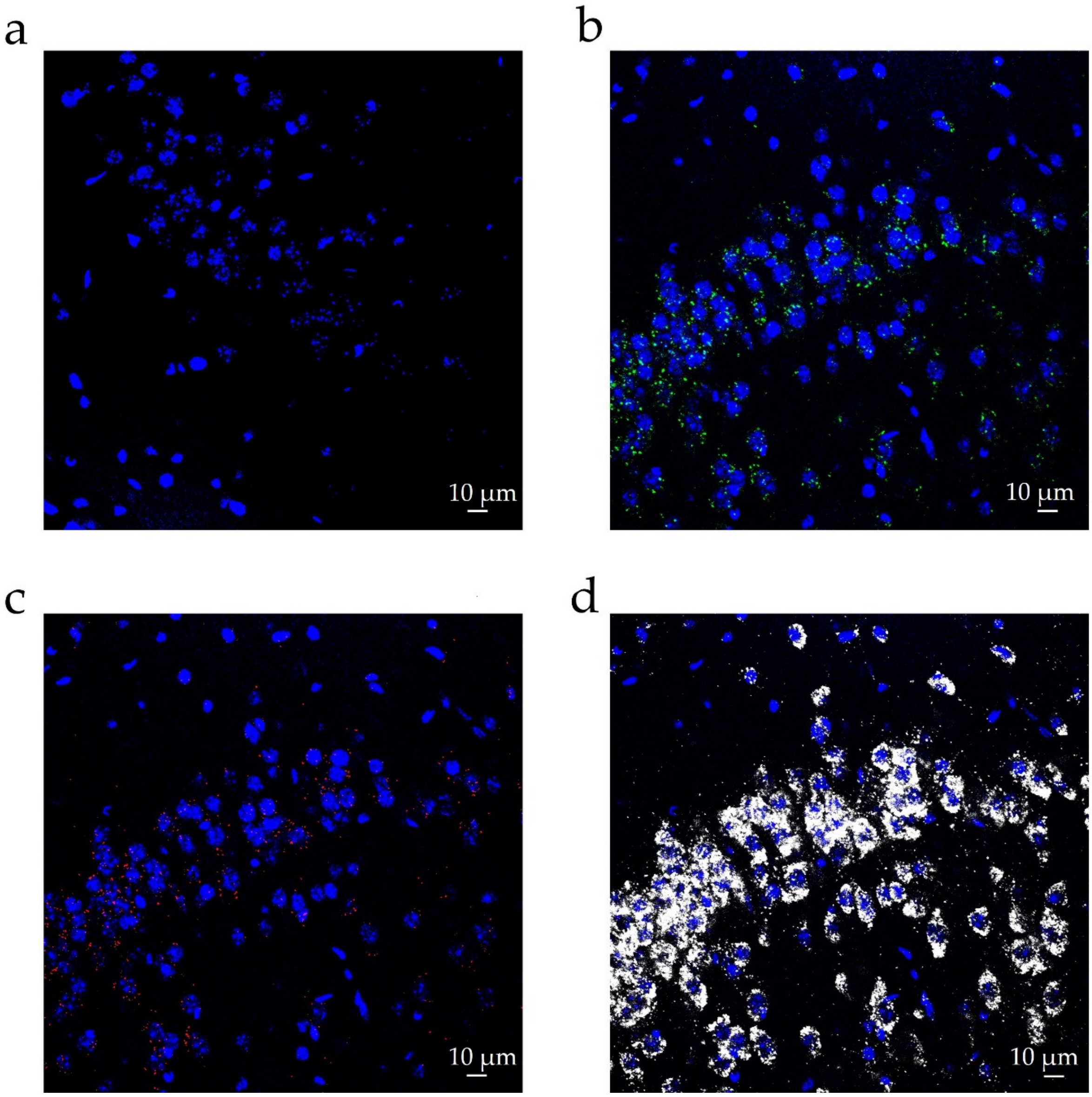

Figure S2. RNAscope triplex positive and negative controls in the mouse piriform cortex. RNAscope triplex negative control probes would hybridize with the bacterial *dabP* gene (**a**). RNAscope triplex positive control probes specific to mouse *Polr2a* (**b**), *Ppib* (**c**) and *Ubc* (**d**). mRNA targets are represented in green, red and white, respectively. Abbrevations: *dabP:* D-box binding PAR BZIP transcription tactor, *Polr2a:* RNA polymerase II subunit A, *Ppib:* peptidylprolyl isomerase B, *Ubc:* ubiquitin C
